# Purification and Characterization of Glucose-6-Phosphate Dehydrogenase from Camel Liver

**DOI:** 10.1155/2014/714054

**Published:** 2014-12-25

**Authors:** Mahmoud A. Ibrahim, Abdel-Hady M. Ghazy, Ahmed M. H. Salem, Mohamed A. Ghazy, Mohamed M. Abdel-Monsef

**Affiliations:** ^1^Molecular Biology Department, National Research Center, Dokki, Cairo 12311, Egypt; ^2^Biochemistry Department, Faculty of Science, Ain Shams University, Cairo 11566, Egypt

## Abstract

Glucose-6-phosphate dehydrogenase from camel liver was purified to homogeneity by ammonium sulfate precipitation and a combination of DEAE-cellulose, Sephacryl S-300 gel filtration, and 2′, 5′ ADP Sepharose 4B affinity chromatography columns. The specific activity of camel liver G6PD is increased to 1.80438 units/mg proteins with 63-fold purification. It turned out to be homogenous on both native PAGE and 12% SDS PAGE, with a molecular weight of 64 kDa. The molecular weight of the native form of camel liver G6PD was determined to be 194 kDa by gel filtration indicating a trimeric protein. The *K*_*m*_ value was found to be 0.081 mM of NADP^+^. Camel liver G6PD displayed its optimum activity at pH 7.8 with an isoelectric point (*p*I) of pH 6.6–6.8. The divalent cations MgCl_2_, MnCl_2_, and CoCl_2_ act as activators; on the other hand, CaCl_2_ and NiCl_2_ act as moderate inhibitors, while FeCl_2_, CuCl_2_, and ZnCl_2_ are potent inhibitors of camel liver G6PD activity. NADPH inhibited camel liver G6PD competitively with *K*_*i*_ value of 0.035 mM. One binding site was deduced for NADPH on the enzyme molecule. This study presents a simple and reproducible purification procedure of G6PD from the camel liver.

## 1. Introduction

Glucose-6-phosphate dehydrogenase (G6PD, EC 1.1.1.49, *β*-D-glucose-6-phosphate; NADP oxidoreductase) is the initial and the key regulatory enzyme in the pentose phosphate pathway [1–4]. It catalyzes the first step of the pentose phosphate pathway (PPP) [5] which is responsible for the production of NADPH and ribose-5-phosphate [6]. G6PD catalyzes the conversion of glucose 6-phosphate to 6-phosphogluconate in the presence of NADP [7–10]. Pentose phosphate pathway has three important functions: (i) serving as the route of entry of pentoses to the glycolytic pathway production of reducing equivalents in the form of NADPH; (ii) production of pentose phosphates necessary for nucleotide biosynthesis; and (iii) production of reducing equivalents in the form of NADPH [5, 11–13].

NADPH is a hydrogen and electron donor for many of metabolic reactions [3], including fatty acid and cholesterol biosynthesis. NADPH is essential for protecting the cell against oxidative stress by transferring its reductive power to oxidized glutathione (GSSG) via glutathione disulfide reductase (GR) [14]. NADPH is also required for maintenance of a reducing balance in cells exposed to high concentrations of oxygen free radicals including erythrocytes, lens, and cornea of the eye and phagocytic cells [15].

G6PD was discovered in 1931 and was isolated in crystalline form by Noltmann and his coworkers from Brewer's yeast [6, 16]. G6PD is found in animal tissues, plants, and microorganisms [11]. In animal tissues, the enzyme is localized in cytosol and mitochondria [17, 18] and in green plants in cytosol and chloroplasts [1, 5, 15]. G6PD is widespread in all tissues and blood cells and is a housekeeping enzyme [19]. G6PD is a significant cytosolic antioxidant enzyme, fundamental for maintenance of cytosolic redox status [20], and plays a critical role in cell growth and death [6, 21].

G6PD deficiency is a common enzymatic defect and leads to hemolysis in the presence of oxidative stress [22]; sufficient quantities of NADPH will not be formed and hydrogen ions will not be available to generate reduced glutathione. As a consequence, oxidative tissue damage may result. Particularly susceptible to oxidative injury is the red blood cell (RBC), as this cell does not have a mechanism other than G6PD of reducing NADP to NADPH [3]. G6PD is purified and identified from several sources including the hyperthermophilic bacterium* Thermotoga maritima* [23],* Schizosaccharomyces pombe* [24], nematodes [25], rabbit liver lumenal endoplasmic reticulum [18], mouse liver [26], dog liver [5], rat brain [27], peroxisomes from guinea pig small intestine [28], bovine lense [1], human placenta [12], buffalo erythrocyte [15], human erythrocyte [29], sheep kidney cortex [6], and rat kidney [30].

This study aims at purifying and characterizing G6PD from the camel liver. The ultimate goal of our study is enzyme production for medical and industrial applications. It is worthwhile to develop economical production method of G6PD from the locally available rich source to establish a continuous supply and quick delivery of different purity grades of the enzyme. Camel is one of the most common domestic mammals in Egypt, Arab world, and the Middle East area. Liver has been selected for the present study since it contains the highest G6PD activity.

## 2. Materials and Methods

### 2.1. Liver Material

Fresh liver samples of camel* Camelus dromedarius* were obtained from a local slaughterhouse and stored at −40°C. Liver samples were collected from at least six different animals.

### 2.2. Chemicals

Glucose-6-phosphate (G6P), *β*-Nicotinamide adenine dinucleotide phosphate (NADP^+^), *β*-Nicotinamide adenine dinucleotide phosphate, reduced (NADPH), 6-phosphogluconic acid (6PG), adenosine-5′-triphosphate (ATP), adenosine diphosphate (ADP), *β*-Nicotinamide adenine dinucleotide (NAD), *β*-Nicotinamide adenine dinucleotide, reduced (NADH), DL-Dithiothreitol (DTT), nitroblue tetrazolium (NBT), phenazine methosulphate (PMS), blue dextran, bovine serum albumin (BSA), Sephacryl S-300, molecular weight marker kits for gel filtration, diethylaminoethyl-cellulose (DEAE-Cellulose), and chemicals for electrophoresis and isoelectric focusing (IEF) standard markers mixture* p*I 3.6–9.3 were purchased from Sigma Chemical Co. Molecular weight SDS marker proteins and 2′, 5′ ADP Sepharose 4B were products of Pharmacia Fine Chemicals Co.

### 2.3. Assay of Glucose-6-Phosphate Dehydrogenase Activity

The G6PD activity assay was carried out according to the method described by Betke et al. [31] by monitoring the NADPH production spectrophotometrically at 340 nm. The assay mixture contained 10 mM MgCl_2_, 0.2 mM NADP^+^, and 0.6 mM G6P in 100 mM Tris/HCl buffer, pH 8.0, and was initiated by addition of the enzyme. One unit (U) of enzyme activity is the amount of enzyme required to reduce 1 *μ*mol of NADP^+^ per minute.

### 2.4. Purification of Glucose-6-Phosphate Dehydrogenase from Camel Liver

#### 2.4.1. Preparation of Crude Extract

Ten grams of liver was minced and homogenized by omnimixer (Sorvall Dupont Instruments), with two volumes (2 mL/gm tissue) of 0.02 M Tris/HCI buffer, pH 7.6, containing 1 mM *β*-mercaptoethanol and 1 mM EDTA on ice. The mixture was centrifuged at 12.000 ×g for 30 min at 4°C and the pellet was discarded. The supernatant containing the enzyme activity was saved and designated the crude extract.

#### 2.4.2. Ammonium Sulfate Precipitation

The crude extract was brought to 40% saturation by addition of solid ammonium sulfate and stirred for 30 min at 4°C and then centrifuged for 30 min at 12.000 ×g. Sufficient solid (NH_4_)_2_SO_4_ was added to the supernatant to give a final saturation of 60%. The pellet was obtained by centrifugation at 12000 ×g for 30 min and dissolved in 0.02 M Tris/HCI buffer, pH 7.6, containing 1 mM *β*-mercaptoethanol and 1 mM EDTA and dialysed against the same buffer and designated ammonium sulfate fraction.

#### 2.4.3. DEAE-Cellulose Column Chromatography

The dialyzed sample was chromatographed on DEAE-cellulose column (12 × 2.4 cm i.d.) previously equilibrated with 0.02 M Tris/HCI buffer, pH 7.6, containing 1 mM *β*-mercaptoethanol and 1 mM EDTA. The adsorbed proteins were eluted with stepwise NaCl gradient ranging from 0 to 1 M prepared in the equilibration buffer at a flow rate of 60 mL/hour and 5 mL fractions were collected. Fractions containing G6PD activity were pooled and lyophilized.

#### 2.4.4. Sephacryl S-300 Column Chromatography

The DEAE-cellulose concentrated solution containing the G6PD activity was applied onto a Sephacryl S-300 column (142 cm × 1.75 cm i.d.). The column was equilibrated and developed with 0.02 M Tris/HCI buffer, pH 7.6, containing 1 mM *β*-mercaptoethanol and 1 mM EDTA at a flow rate of 30 mL/hour and 2 mL fractions were collected and the fractions containing G6PD activity were pooled and lyophilized.

#### 2.4.5. Chromatography on 2′, 5′ ADP Sepharose 4B Affinity Column

The Sephacryl S-300 concentrated fractions containing the G6PD activity were applied onto a 2′, 5′ ADP Sepharose 4B affinity column (3 cm × 2.4 cm i.d.) equilibrated with 0.02 M Tris/HCI buffer, pH 7.6, containing 1 mM *β*-mercaptoethanol and 1 mM EDTA (Buffer A). The column was washed with the same buffer at a flow rate of 15 mL/h to remove all the nonspecifically bound proteins. The G6DP was eluted with buffer A containing 0.5 mM NADP^+^.

### 2.5. Electrophoretic Analysis

Native gel electrophoresis was carried out using 7% PAGE according to Smith [32]. SDS-PAGE was performed with 12% polyacrylamide gel according to Laemmli [33]. The subunit molecular weight of the purified camel liver G6PD enzyme was determined using SDS-PAGE as described by Weber and Osborn [34]. Electrofocusing was performed according to O'Farrell [35] and the isoelectric point (*p*I) values were calculated from a calibration curve as described by Ubuka et al. [36]. Proteins were stained with 0.25% coomassie brilliant blue R-250.

### 2.6. Protein Quantification

Protein was quantified by the dye binding assay method of Bradford [37] using BSA as a standard protein.

## 3. Results

### 3.1. Purification of Glucose-6-Phosphate Dehydrogenase from Camel Liver

**Figure 1:**
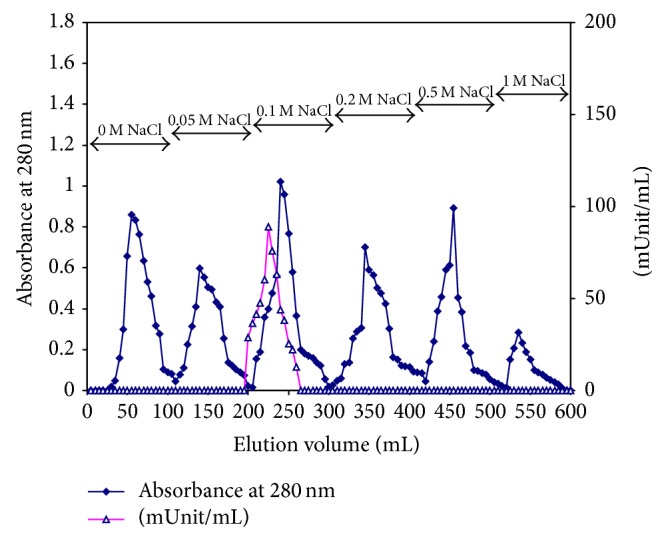
A typical elution profile for the chromatography of the camel liver ammonium sulfate fraction on DEAE-cellulose column (12 cm × 2.4 cm) previously equilibrated with 0.02 M Tris/HCI buffer, pH 7.6 containing 1 mM *β*-mercaptoethanol, and 1 mM EDTA. The proteins were eluted by a stepwise gradient of NaCl ranging from 0 to 1 M in the equilibration buffer. 5 mL fractions were collected at a flow rate of 60 mL/h.

**Figure 2:**
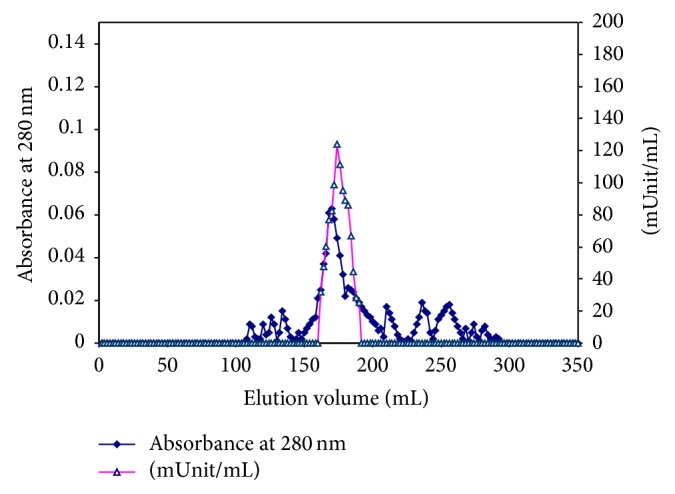
A typical elution profile for the chromatography of the concentrated pooled DEAE-cellulose fractions containing (camel liver G6PD) activity on Sephacryl S-300 column (142 cm × 2.4 cm) previously equilibrated with 0.02 M Tris/HCI buffer, pH 7.6 containing 1 mM *β*-mercaptoethanol, and 1 mM EDTA. The proteins were eluted by the same buffer. 2 mL fractions were collected at a flow rate of 30 mL/h.

**Figure 3:**
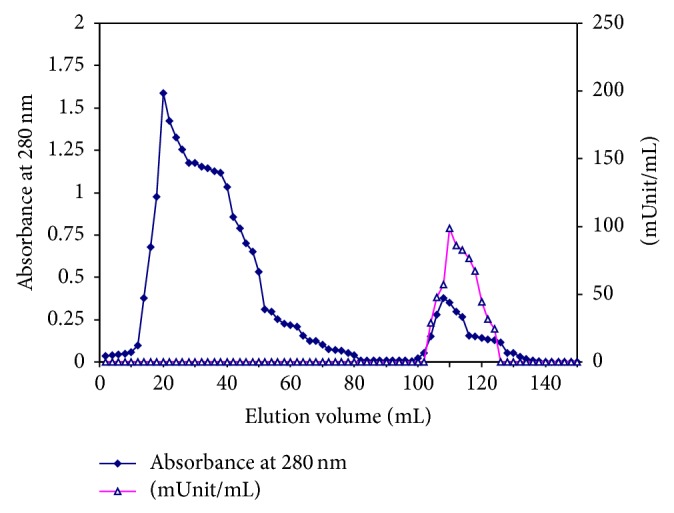
A typical elution profile for the chromatography of the concentrated pooled Sephacryl S-300 column fractions containing (camel liver G6PD) activity on 2′, 5′ ADP Sepharose 4B column (3 cm × 2.4 cm) previously equilibrated with 0.02 M Tris/HCI buffer, pH 7.6 containing 1 mM *β*-mercaptoethanol, and 1 mM EDTA (buffer A). The proteins were eluted by the same buffer containing 0.5 mM NADP^+^. 2 mL fractions were collected at a flow rate of 15 mL/h.

The G6PD specific activity of camel liver crude extract was found to be 0.0286 unit/mg protein. A typical purification scheme of G6PD from the camel liver is presented in Table 1. After ammonium sulfate precipitation, most of the glucose-6-phosphate dehydrogenase activity was precipitated; 85.97% of the activity was recovered. The DEAE-cellulose elution profile (Figure 1) revealed the presence of one major peak containing G6PD activity eluted with 0.1 M NaCl and designated camel liver G6PD. The DEAE-cellulose fractions were pooled, concentrated by lyophilization, and applied onto a Sephacryl S-300 column. The elution profile of camel liver G6PD on the Sephacryl S-300 column (Figure 2) revealed the presence of one peak of the enzyme activity. The specific activity of CLG6PD was increased to 0.26647 units/mg protein, which represent 9.317-fold purification over the crude extract with 58.01% yield. The Sephacryl S-300 fractions were pooled, concentrated by lyophilization, and applied onto 2′, 5′ ADP Sepharose 4B Affinity column (Figure 3). The specific activity of camel liver G6PD was increased to 1.80438 units/mg protein, which represent 63.09-fold purification over the crude extract with 49.61% yield (Table 1).

### 3.2. Molecular Weight Determination by Gel Filtration

Camel liver G6PD was eluted at 176 mL from Sephacryl S-300 column and its native molecular weight was deduced to be 194 ± 2.3 kDa.

### 3.3. Electrophoretic Analyses of Camel Liver G6PD

**Figure 4:**
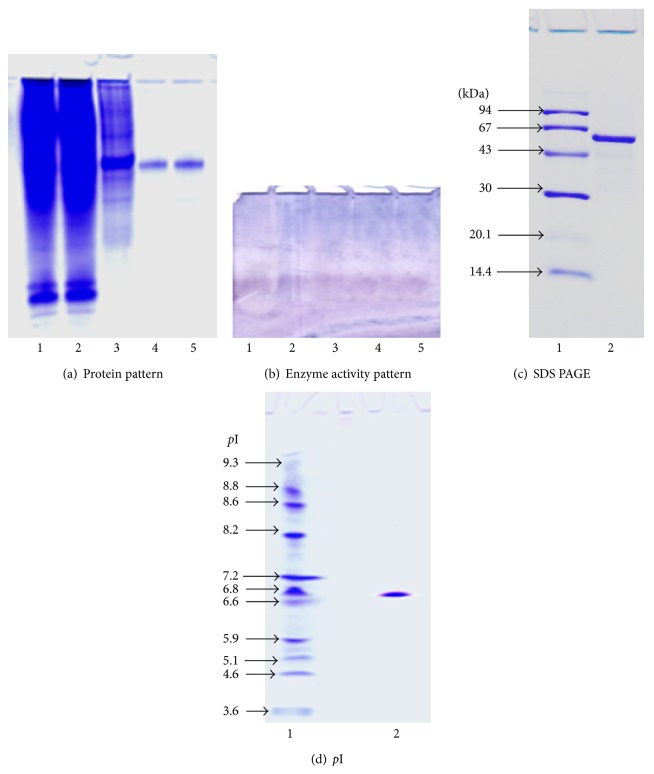
Electrophoretic analysis of (camel liver G6PD) the different purification steps on 7% native polyacrylamide gel. (a) Protein pattern: (1) crude extract, (2) ammonium sulfate fraction, (3) 0.1 M DEAE-cellulose fraction, (4) Sephacryl S-300 purified fraction, and (5) 2′, 5′ ADP Sepharose 4B fractions. (b) Enzyme pattern: (1) crude extract, (2) ammonium sulfate fraction, (3) 0.1 M DEAE-cellulose fraction, (4) Sephacryl S-300 purified fraction, and (5) 2′, 5′ ADP Sepharose 4B fractions. (c) Subunit molecular weight determination by electrophoretic analysis of camel liver G6PD on 12% SDS-polyacrylamide gel: (1) molecular weight marker proteins and (2) purified camel liver glucose-6-phosphate dehydrogenase enzyme camel liver G6PD. (d) Isoelectrofocusing: (1) isoelectric point (*p*I) marker proteins and (2) the purified camel liver glucose-6-phosphate dehydrogenase camel liver G6PD.

Samples from the different purification steps, crude extract, ammonium sulfate fraction, DEAE-cellulose fraction, Sephacryl S-300 fraction, and 2′, 5′ ADP Sepharose 4B fraction of camel liver G6PD were analyzed electrophoretically on 7% native PAGE (Figure 4(a)). Single protein bands coincided with the enzyme activity bands indicating the tentative purity of the enzyme preparation (Figure 4(b)). Electrophoretic analysis of denatured purified camel liver G6PD enzyme on SDS-PAGE was compared with molecular weight marker proteins; the subunit molecular weight was calculated to be 64 ± 1.48 kDa (Figure 4(c)). The purified camel liver G6PD was electrofocused and the isoelectric point (*p*I) value was calculated. Camel liver G6PD enzyme showed a single molecular species with* p*I value of 6.6–6.8 (Figure 4(d)).

### 3.4. Determination of Optimum pH

**Figure 5:**
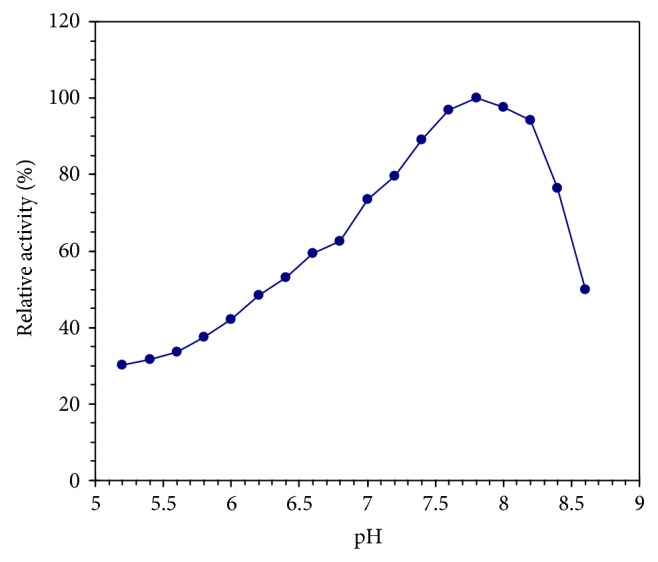
Effect of pH on camel liver G6PD using 0.02 M Tris/HCI buffer of various pH values.

The effect of pH on the camel liver G6PD activity was examined in 0.02 M Tris/HCI buffer, pH (5.2–8.8). The pH profile of camel liver G6PD displayed its optimum activity at pH 7.8 (Figure 5).

### 3.5. Effect of Divalent Cations

**Figure 6:**
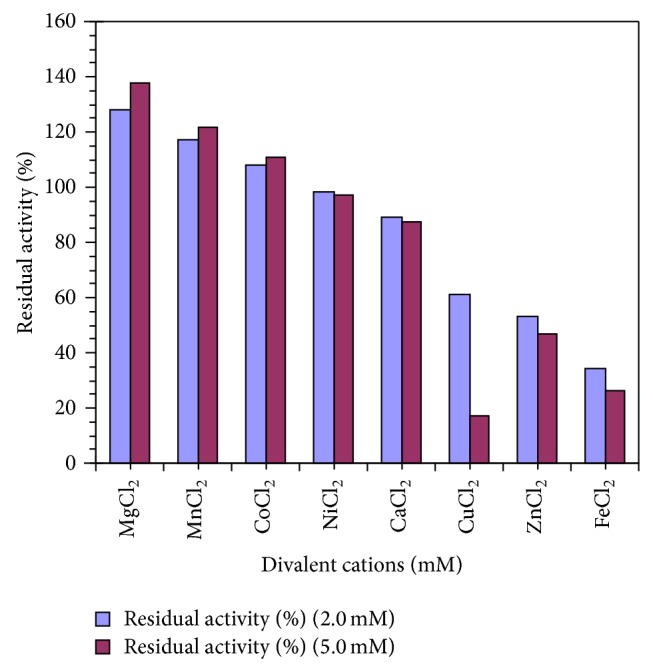
Effect of divalent cations on the purified camel liver G6PD.

The purified camel liver G6PD was preincubated with 2 mM and 5 mM of each cation at 37°C and the activity was assayed. A control test without any cation was taken as 100% relative activity. The activity of the camel liver G6PD is increased about 1.28-, 1.17-, and 1.07-fold in the presence of 2 mM MgCl_2_, MnCl_2_, and CoCl_2_, respectively, and increased about 1.37-, 1.21-, and 1.10-fold in the presence of 5 mM MgCl_2_, MnCl_2_, and CoCl_2_, respectively. In contrast, CaCl_2_ and NiCl_2_ were found to be moderate inhibitors of camel liver G6PD activity while FeCl_2_, CuCl_2_, and ZnCl_2_ are potent inhibitors of camel liver G6PD (Figure 6).

### 3.6. Michaelis-Menten Constant (*K*_*m*_) Value

**Figure 7:**
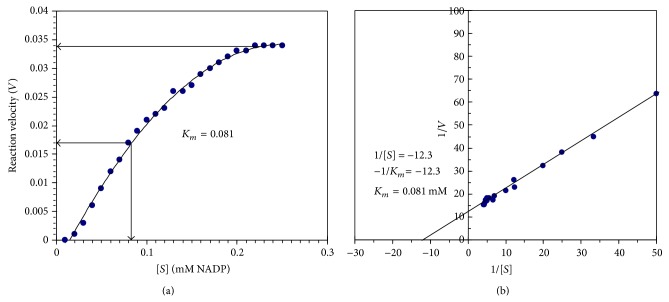
(a) Effect of the substrate *β*-Nicotinamide adenine dinucleotide phosphate (NADP^+^) concentration in mM on the reaction velocity of camel liver G6PD. The reaction velocity is the change in absorbance at 340 nm per 60 min. (b) Lineweaver-Burk plot relating the reciprocal of the reaction velocity of camel liver G6PD to (NADP^+^) concentration in mM.

The *K*_*m*_ value of camel liver G6PD was found to be 0.081 mM NADP^+^ and the corresponding maximum velocity (*V*_max⁡_) was calculated to be 1875.86 munits/mg protein (Figure 7(a)). For further confirmation, a Lineweaver-Burk plot for the reciprocal of the reaction velocity (1/*V*) and substrate concentration (1/[*S*]) was constructed; the *K*_*m*_ value of camel liver G6PD was deduced to be 0.081 mM NADP^+^ (Figure 7(b)).

### 3.7. Effect of Various Inhibitors

**Figure 8:**
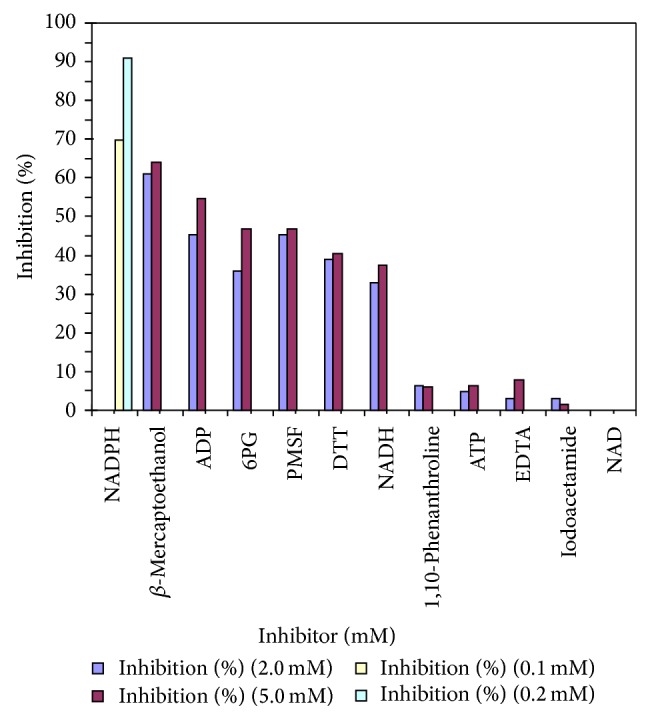
Effect of inhibitors on purified camel liver G6PD.

The purified camel liver G6PD is preincubated with 2 mM and 5 mM of each inhibitor (0.1 mM and 0.2 mM of NADPH) for 5 min at 37°C and the residual activity was calculated as a ratio of a control lacking inhibitor. NADPH is found to be the most potent inhibitor of the camel liver G6PD activity (Figure 8).

### 3.8. Kinetics of Camel Liver G6PD Inhibition by NADPH

**Figure 9:**
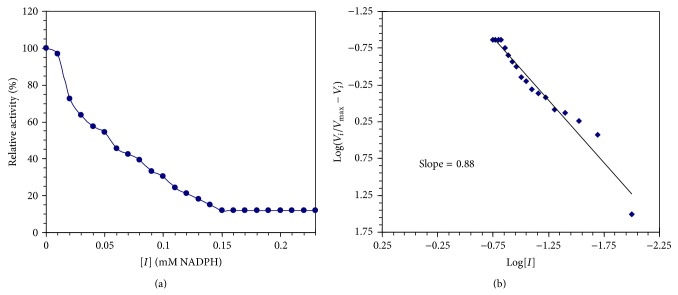
(a) Inhibition of camel liver G6PD by varying concentrations of NADPH. (b) Hill plot for inhibition of camel liver G6PD by varying concentrations of NADPH where *V*_max⁡_ is the enzyme activity in absence of inhibitor. *V*_*i*_ is the enzyme activity in presence of inhibitor and [*I*] is inhibitor concentration in mM of NADPH.

**Figure 10:**
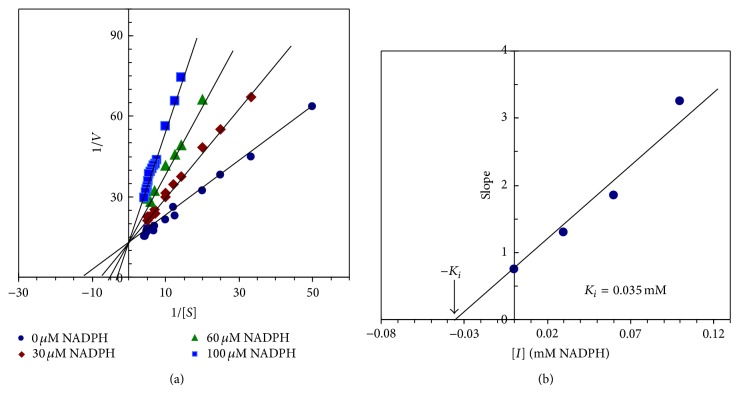
(a) Lineweaver-Burk plots showing the type of inhibition of camel liver G6PD by NADPH. The activity of camel liver G6PD was measured with varying concentrations of the substrate (NADP^+^) in absence and presence of three various concentrations of NADPH. (b) Determination of the inhibition constant (*K*_*i*_) value for the inhibition of camel liver G6PD by NADPH. The plotted slope values were determined from the lines of the reciprocal plots of the different inhibitor concentrations.

The NADPH exhibited the most potent inhibitory effect on the camel liver G6PD. The effect of varying concentrations of NADPH on the camel liver G6PD activity presented in (Figure 9(a)). A maximum inhibition of camel liver G6PD by NADPH was found to be 87.88% at 0.17 mM of NADPH. In the Hill plot, when log *V*_*i*_/*V*_max⁡_ − *V*_*i*_ values were plotted against log [*I*] values of the NADPH, a straight line was obtained with slope of about 0.88 for camel liver G6PD indicating the presence of one binding site for NADPH in camel liver G6PD (Figure 9(b)). The type of inhibition of camel liver G6PD by NADPH is competitive type since the presence of the inhibitor did not alter the *V*_max⁡_ value but it increases the *K*_*m*_ value (Figure 10(a)). The *K*_*i*_ value of the camel liver G6PD inhibition by NADPH is determined to be 0.035 mM (Figure 10(b)).

## 4. Discussion

G6PD plays a significant role in maintaining the level of NADPH and in generating pentose phosphates for nucleotide biosynthesis [2, 38]. In this study, a simple and reproducible purification method for G6PD from camel liver was used. Camel is one of the most common domestic mammals in Egypt, Arab world, and the Middle East area. The purification procedure was carried out by ammonium sulfate precipitation, ion exchange chromatography on DEAE-cellulose column, gel filtration chromatography on Sephacryl S-300 column, and affinity chromatography on 2′, 5′ ADP Sepharose 4B column. Similar purification procedures of G6PDs were reported from mouse liver [26], from dog liver [5], from bovine lens [1], from human placental [12], from human erythrocyte [29], from sheep kidney cortex [6], and from rat kidney [30].

The chromatography on the DEAE-cellulose column revealed that the camel liver G6PD is moderate, negatively charged, and moderately bound to the DEAE-cellulose matrix since it was eluted with 0.1 M NaCl (Figure 1). The camel liver G6PD was eluted from the Sephacryl S-300 column as a single enzyme activity peak and the deduced molecular mass was found to be 194 ± 2.3 kDa (Figure 2). The camel liver G6PD was eluted from the 2′, 5′ ADP Sepharose 4B column as a single enzyme activity peak (Figure 3). The specific activity of camel liver G6PD was increased to 1.80438 units/mg protein, which represent 63.09-fold purification over the crude extract with 49.61% yield. A large variety of purification fold and recovery percent for G6PD were reported: mouse liver with 8.7% yield [26], dog liver with 18% yield [5], bovine lens 19.7-fold with 13.7% yield [1], human placenta with 62% yield [12], human erythrocyte 91.50-fold with 43% yield [29], and sheep kidney cortex 13.84-fold with 16.96% yield [6].

The purity of camel liver G6PD eluted from the 2′, 5′ ADP Sepharose 4B column was investigated by analysis on 7% native PAGE. Camel liver G6PD turned out to be homogeneous preparation indicating the tentative purity of the enzyme; also the protein band coincided with the enzyme activity band confirming that the single protein band is the enzyme band (Figures 4(a) and 4(b)). The subunit molecular weight of camel liver G6PD was determined by SDS-PAGE to be 64 ± 1.48 kDa (Figure 4(c)). Comparison of subunit molecular weight with that of native intact protein determined by gel filtration indicated that camel liver G6PD is trimeric protein composed of three identical subunits. Distinct G6PD molecular weights were reported from different organism, tissues, and organs. Many G6PDs were reported to have a monomeric structure with subunit molecular weight of 62 kDa as that of bovine lens [1], 54 kDa of human placenta [12], and 52 kDa of dog liver [5]. A dimeric structure was reported for G6PD from rat kidney with a native molecular mass of 144 kDa and subunit molecular mass of 68 kDa [30]. A tetrameric structure was reported for G6PD from mouse liver with a native and subunit molecular weight of 117 and 31 kDa, respectively [26].

The isoelectric point (*p*I) value was estimated for the purified camel liver G6PD at 6.6–6.8 (Figure 4(d)). Lower isoelectric points (*p*I) value was reported for G6PD from bovine lens at 5.14 [1]. The camel liver G6PD displayed the optimum pH at 7.8 (Figure 5). Similarly, the pH optimum was determined to be 7.8 for G6PD from human placenta [12]. Dog liver G6PD had a pH optimum of 7.8 [5]. The optimum pH of the lamb kidney cortex G6PD was determined to be between pH 7.6 and 7.8 [39]. The optimum pH of the sheep kidney cortex G6PD was determined to be pH 7.4 [6].

FeCl_2_, CuCl_2_, CoCl_2_, and ZnCl_2_ inhibited the activity of camel liver G6PD while MnCl_2_ and MgCl_2_ increased the activity of the camel liver G6PD (Figure 6). These results are in accordance with G6PD of rainbow trout liver (*Oncorhynchus mykiss*) which is inhibited by FeCl_2_, CuCl_2_, and ZnCl_2_ [40].

The *K*_*m*_ value of the purified camel liver G6PD was found to be 0.081 mM *β*-Nicotinamide adenine dinucleotide phosphate (NADP^+^) with a maximum velocity of 1875.86 munits/mg protein (Figure 7) indicating the high affinity of the purified G6PD toward the NADP^+^. This value is considerably close to G6PD from rat liver, since *K*_*m*_ value was found to be 0.100 mM NADP^+^. Different *K*_*m*_ values were reported; *K*_*m*_ value was found to be 0.025 mM NADP for G6PD of rat kidney cortex [41], that of bovine lens was 0.008 mM [1], that of human placenta was 0.020 mM [12], that of dog liver was 0.010 mM [5], and that of sheep kidney cortex was 0.0147 mM [6].

In this study, the effect of different specific and characteristic inhibitors on the camel liver G6PD is presented in (Figure 8). NADPH is found to be the most potent inhibitor of the activity of camel liver G6PD since 0.1 mM NADPH inhibited the camel liver G6PD activity 69.70% while 0.2 mM NADPH inhibited the camel liver G6PD activity 90.91%. 6-Phosphogluconic acid, ATP, ADP, and NADH were found to be weak inhibitors of camel liver G6PD activity. NAD has no inhibitory effect on the activity of camel liver G6PD. Similarly, G6PD from mouse liver [26], bovine lens [1], human placenta [12], dog liver [5], human erythrocyte [29], sheep kidney cortex [6], and rat kidney [30] were inhibited by NADPH.

The effect of NADPH concentration on the purified camel liver G6PD indicated that a maximum inhibition of camel liver G6PD by NADPH was found to be 87.88% at 0.17 mM of NADPH (Figure 9(a)). However, full explanation of these data required knowledge of the number of inhibitor molecules bound per enzyme molecule. A linear relationship was observed by constructing the Hill plot for the inhibition of the purified camel liver G6PD by NADPH (Figure 9(b)). The slope of the Hill plot was found to be 0.88 for camel liver G6PD indicating the existence of one binding site for NADPH on camel liver G6PD. The type of inhibition of the purified camel liver G6PD by NADPH was found to be competitive (Figure 10(a)), where the presence of NADPH did not alter the *V*_max⁡_ value but increased the *K*_*m*_ value. For the determination of the *K*_*i*_ value, the slopes of the reciprocal plots lines (Figure 10(a)) were plotted against the NADPH concentration (Figure 10(b)). The *K*_*i*_ value of the enzyme inhibition by NADPH is determined to be 0.035 mM directly from the intercept of the *x*-axis of the plot. Similarly, the *K*_*i*_ value of the G6PD from bovine lens was found to be 0.017 mM NADPH [1] and from dog liver 0.012 mM [5].

*β*-Mercaptoethanol and dithiothreitol inhibited the camel liver G6PD indicating that SH groups in the active site play an important role for camel liver G6PD activity. Phenyl methyl sulfonyl fluoride inhibited camel liver G6PD indicating that serine residue is involved in the active site of the enzyme (Figure 8).

In conclusion, this study presents a simple and convenient method for the purification of glucose-6-phosphate dehydrogenase from camel liver as a rich source. The method is adaptable for large amounts of production of the enzymes by scaling up from the laboratory level to the larger scales. The characterization of the purified glucose-6-phosphate dehydrogenase established the optimum conditions for the activity of this enzyme and will allow their uses in various applications with maximum efficiency.

## Figures and Tables

**Table 1:** A typical purification scheme of camel liver G6PD.

Purification steps^*^	Total protein (mg)	Total activity (unit^†^)	Specific activity^‡^	Yield (%)	Fold purification
Crude extract	156.28	4.4801	0.0286	100	1.00
Ammonium sulphate fraction	49.530	3.8517	0.0777	85.97	2.719
DEAE-cellulose					
Camel liver G6PD (0.1 M NaCl)	18.032	3.0527	0.16929	68.13	5.919
Sephacryl S-300					
Camel liver G6PD	9.7562	2.5990	0.26647	58.01	9.317
2′, 5′ ADP Sepharose 4B					
Camel liver G6PD	1.2320	2.2230	1.80438	49.61	63.09

^*^All data is based on 10 gm camel liver.

^†^One unit of glucose-6-phosphate dehydrogenase enzyme activity is identified as the amount of enzyme required to reduce 1 *μ*mol of NADP^+^ per minute.

^‡^Specific activity is expressed as units/mg protein.

## Abbreviations

BSA: Bovine serum albumin
PAGE: Polyacrylamide gel electrophoresis
NBT: Nitroblue tetrazolium
PMS: Phenazine methosulphate
PPP: Pentose phosphate pathway
${\text{NADP}}^{+}$: *β*-Nicotinamide adenine dinucleotide phosphate
NADPH: Reduced *β*-Nicotinamide adenine dinucleotide phosphate
G6PD: Glucose-6-phosphate dehydrogenase.
